# Histidine oxidation in lytic polysaccharide monooxygenase

**DOI:** 10.1007/s00775-023-01993-4

**Published:** 2023-02-25

**Authors:** Magne Torbjörnsson, Marlisa M. Hagemann, Ulf Ryde, Erik Donovan Hedegård

**Affiliations:** 1 Department of Theoretical Chemistry, Lund University, Chemical Centre, P. O. Box 124, 221 00 Lund, Sweden; 2 Department of Physics, Chemistry, and Pharmacy, University of Southern Denmark, Campusvej 55, 5230 Odense M, Denmark

**Keywords:** Lytic polysaccharide monooxygenase, Histidine oxidation, Histidine methylation, QM/MM

## Abstract

The lytic polysaccharide monooxygenases (LPMOs) comprise a super-family of copper enzymes that boost the depolymerisation of polysaccharides by oxidatively disrupting the glycosidic bonds connecting the sugar units. Industrial use of LPMOs for cellulose depolymerisation has already begun but is still far from reaching its full potential. One issue is that the LPMOs self-oxidise and thereby deactivate. The mechanism of this self-oxidation is unknown, but histidine residues coordinating to the copper atom are the most susceptible. An unusual methyl modification of the NE2 atom in one of the coordinating histidine residues has been proposed to have a protective role. Furthermore, substrate binding is also known to reduce oxidative damage. We here for the first time investigate the mechanism of histidine oxidation with combined quantum and molecular mechanical (QM/MM) calculations, with outset in intermediates previously shown to form from a reaction with peroxide and a reduced LPMO. We show that an intermediate with a [Cu–O]^**+**^ moiety is sufficiently potent to oxidise the nearest C–H bond on both histidine residues, but methylation of the NE2 atom of His-1 increases the reaction barrier of this reaction. The substrate further increases the activation barrier. We also investigate a [Cu–OH]^2**+**^ intermediate with a deprotonated tyrosine radical. This intermediate was previously proposed to have a protective role, and we also find it to have higher barriers than the corresponding a [Cu–O]^**+**^ intermediate.

**Graphical abstract:**

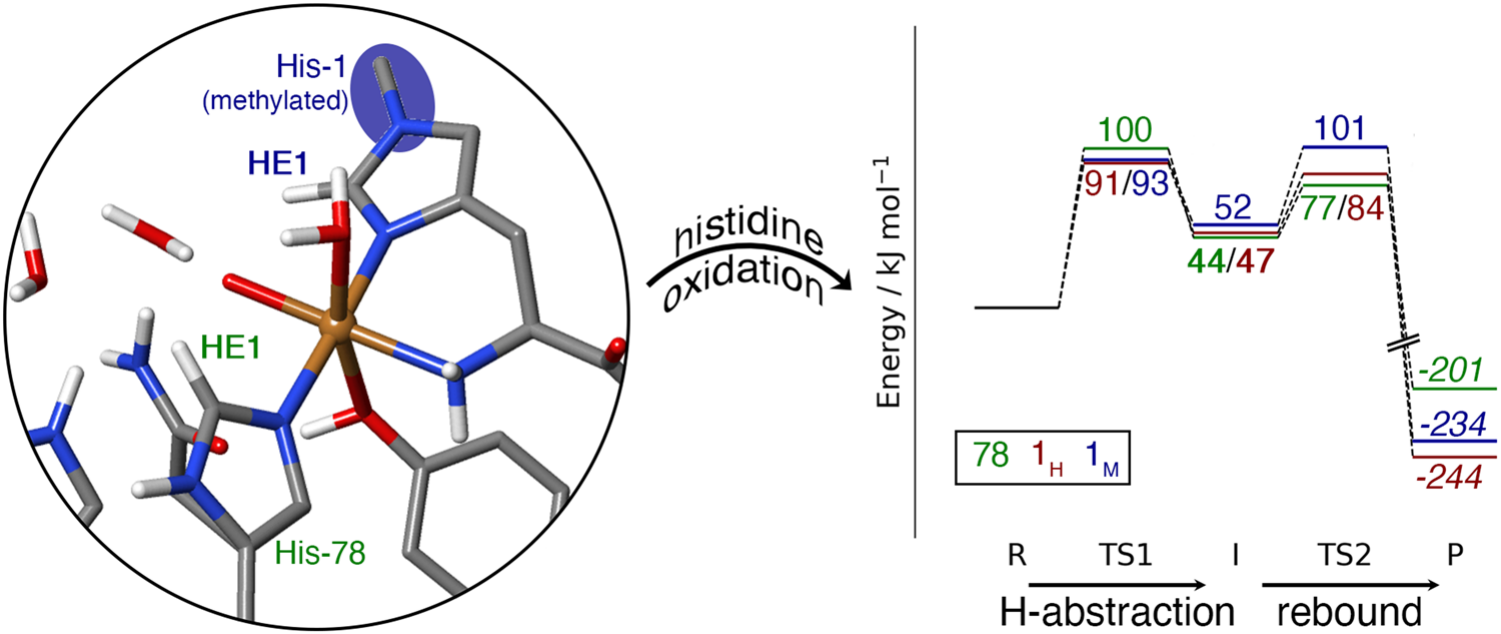

## Introduction

The copper-dependent lytic polysaccharide monooxygenases (LPMOs) comprise a super-family of enzymes, categorised as auxiliary activity (AA) enzymes with the distinct members AA9–AA17 (AA12 is exempted) [1–9]. The LPMOs boost depolymerisation of polysaccharides such as cellulose and chitin [1, 2, 10–13], which has attracted considerable attention considering that the cellulose biopolymer is the largest component of biomass waste [14]. However, the remarkable recalcitrance of the cellulose and other polysaccharides has so far prevented cost-efficient up-cycling of biomass waste to higher-value products. The LPMOs bring hope that such up-cycling may become possible.

The boost in polysaccharide degradation stemming from LPMOs is due to an oxidative reaction on the polysaccharide substrate. Originally, the oxidation was believed to progress with O_2_ as the co-substrate [1], but more recent studies have shown that H_2_O_2_ leads to significantly faster reactions [15–18]. The nature of the true co-substrate in nature is still debated [19], but H_2_O_2_ can be employed as co-substrate. In fact, for *Lentinus similis* LPMO (*Ls*AA9), a recent study could not detect any O_2_ activation in the presence of substrate, whereas H_2_O_2_ led to fast scission of the glycoside bond [20].

**Fig. 1:**
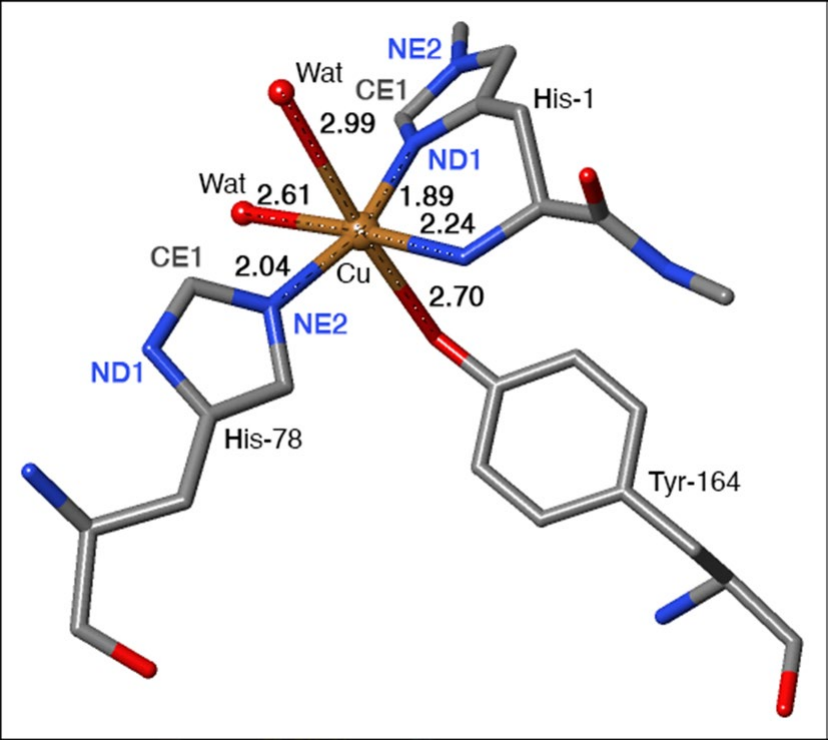
The active site of LPMO, based on the 5N04 crystal structure of *Ls*AA9 without substrate [21], showing the Cu ion in the Cu(II) resting state with six ligands

The active site of the LPMOs consists of a copper ion ligated to three nitrogen donor atoms: one histidine ligand binds by the imidazole group, while the other histidine ligand is the N-terminal and binds both by the imidazole group and the amino-terminal –NH_2_ group [3]. This has been called the histidine brace motif and it is shown in Fig. 1.

Industrial use of LPMOs for cellulose depolymerisation [22] has already begun, but we are still far from reaching the full potential. One issue is that the LPMOs self-oxidise and thereby deactivate. A significant step in the direction of better utilisation of LPMOs could be achieved by elucidation of the molecular mechanism for this deactivation. So far, most theoretical studies of the LPMO molecular mechanism have focused on either the substrate binding process [23–25], or the reaction with the bound substrate [26–31]. In the latter case, the theoretical investigations have shown that the reaction with the substrate requires a very strong oxidant and a copper(II)–oxyl species has been the preferred one. It has also been shown that this oxyl can be formed from H_2_O_2_ [29–31]. Very recently, theoretical as well as experimental investigations have been carried out for the reaction of H_2_O_2_ and LPMOs (with copper reduced) without substrate [32–35]. These investigations have attempted to trap and spectroscopically characterise potential intermediates relevant for substrate- or self-oxidation. Several such intermediates have been identified; one of these contains a [Cu–OH]^2+^ moiety formed by extracting the hydrogen for the OH group of the coordinating tyrosine (Tyr-164 in Fig. 1), thus leaving a tyrosine radical.

Yet, there exist currently no investigations of the actual oxidative damage. It has been noted to occur on the histidine residues coordinating copper [17], but the oxidation products and mechanism are unknown. Here, we carry out the first investigations of the initial steps of the oxidation in *Ls*AA9. We focus on the two coordinating histidine residues. Since the substrate is known to protect from oxidative damage [17], we investigate both substrate-bound and substrate-free *Ls*AA9. Interestingly, an investigation by Petrovic et al. [36] showed that, in addition to the substrate, a methylation of the NE2 atom [3] of the N-terminal, coordinating histidine also protects from auto-oxidative inactivation (cf. Fig. 1). This methylation is seen in many fungal LPMOs, but its function has remained unknown, since non-methylated enzymes are also catalytically active [37]. While the findings in Ref. [36] may suggest a protective role, a mechanistic explanation of the results are lacking. We have therefore included both a NE2-methylated and a non-methylated *Ls*AA9 in this investigation.

## Methods

### QM calculations

All QM calculations were performed with the Turbomole software (version 7.5.1) [38]. We employed two DFT methods, TPSS [39] and B3LYP [40–42], and two basis sets, def2-SV(P) and def2-TZVPD [43, 44]. The calculations were sped up by expanding the Coulomb interactions in an auxiliary basis set, the resolution-of-identity (RI) approximation [45, 46]. Empirical dispersion corrections were included with the DFT-D3 approach [47] and Becke–Johnson damping [48], as implemented in Turbomole.

The QM calculations for triplet states were performed with an unrestricted formalism, whereas the singlet states were obtained both with restricted and unrestricted formalisms. In the latter case, the occupation of alpha and beta orbitals was defined to be equal, which corresponds to a broken-symmetry (BS) approach [49]. The molecular orbitals from a converged triplet state were employed as starting point. The nature of the electronic states was verified and described by examining Mulliken spin populations.

### QM/MM calculations

The QM/MM calculations were performed with the ComQum software [50, 51]. In this approach, the protein and solvent are split into two subsystems: System 1 (the QM region) was relaxed by QM methods. System 2 contained the remaining part of the protein and the solvent, and it was kept fixed at the original coordinates (equilibrated crystal structure) to avoid the risk that different calculations end up in different local minima.

In the QM calculations, system 1 was represented by a wavefunction, whereas all the other atoms were represented by an array of partial point charges, one for each atom, taken from the MM setup. Thereby, the polarisation of the QM system by the surroundings is included in a self-consistent manner (electrostatic embedding). When there is a bond between systems 1 and 2 (a junction), the hydrogen link-atom approach was employed: The QM system was capped with hydrogen atoms (hydrogen link atoms, HL), the positions of which are linearly related to the corresponding carbon atoms (carbon link atoms, CL) in the full system [50, 52]. All atoms were included in the point-charge model, except the CL atoms [53].

The total QM/MM energy in ComQum was calculated as [50, 51]

1 $${E}_{\text{QM/MM}}={E}_{{\text{QM1}}+{\rm ptch}2}^{\text{HL}}+{E}_{{\text{MM}}12,{q}_{1}= {0}} ^{\text{CL}}-{E}_{{\text{MM1}},{q}_{1}= {0}} ^{\text{HL}},$$

where ${E}_{{{\text{QM1}}+{\rm ptch2}}}^{{\text{HL}}}$ is the QM energy of the QM system truncated by HL atoms and embedded in the set of point charges modelling system 2 (but excluding the self-energy of the point charges). ${E}_{{\text{MM1}},{q}_{1}=0}^{\text{HL}}$ is the MM energy of the QM system, still truncated by HL atoms, but without any electrostatic interactions. Finally, ${E}_{{\text{MM12,}}{q}_{1}= {0} }^{\text{CL}}$ is the classical energy of all atoms in the system with CL atoms and with the charges of the QM region set to zero (to avoid double-counting of the electrostatic interactions). Thus, ComQum employs a subtractive scheme with electrostatic embedding and van der Waals link-atom corrections [54]. No cutoff is used for any of the interactions in the three energy terms in Eq. (2).

The geometry optimisations were performed at the TPSS/def2-SV(P) level of theory with a convergence criterium of 10^–6^ a.u. for energies and 10^–3^ a.u. for the maximum norm of the Cartesian gradients. After convergence, single-point QM energy calculations with the point-charge model were performed with the def2-TZVPD basis set and either the TPSS or B3LYP methods. Owing to convergence problems, B3LYP calculations with the substrate had to be done with the def2-SV(P) basis set and they were extrapolated to the def2-TZVPD basis set by TPSS calculations:

2 $${E}_{\text{QM/MM}}(\text{B3/TZ)}= {E}_{\text{QM/MM}}(\text{B3/SV)}+ {E}_{{\text{QM1}}+{\rm ptch}2}^{\text{HL}}\left(\text{TP/TZ}\right)-{E}_{{\text{QM1}}+{\rm ptch}2}^{\text{HL}}\left(\text{TP/SV}\right),$$

where ${E}_{\text{QM/MM}}(\text{B3/SV)}$ is the QM/MM energy obtained at the B3LYP/def2-SV(P) level, ${E}_{{\text{QM1}}+{\rm ptch}2}^{\text{HL}}(\text{TP/TZ})$ is the QM energy of system 1 in a point-charge surrounding (first term on the right-hand-side of Eq. 1) at the TPSS/def2-TZVPD level and ${E}_{{\text{QM}}1+\text{ptch2}}^{\text{HL}}(\text{TP/SV})$ is the corresponding term with the def2-SV(P) basis set. In the main text, only the extrapolated B3LYP-D3/def2-TZVPD results are discussed.

In general, increasing the basis set from def2-SV(P) to def2-TZVPD changes the calculated energies by 10 kJ/mol on (absolute) average, with a varying sign. Changing the functional to TPSS has a restricted effect on the triplet energies (8 kJ/mol mean absolute difference). However, TPSS systematically stabilises the singlet states, especially the closed-shell singlet, by 44 kJ/mol on average. TPSS sometimes gives a spurious broken-symmetry state, intermediate between the closed-shell and open-shell singlet, with a spin distribution similar to that of the open-shell singlet, but with 2–8 times lower magnitude. For some states, we were unable to find a proper open-shell singlet with TPSS. Therefore, we have based the discussion on the B3LYP energies and in some cases, we used geometries optimised with B3LYP/def2-SV(P).

The setup of the protein with and without the substrate has been described before and we refer to Ref. [35] for details. The total system was spherical and non-periodic with 24,186 or 24,243 atoms (without or with the substrate, respectively).

**Fig. 2:**
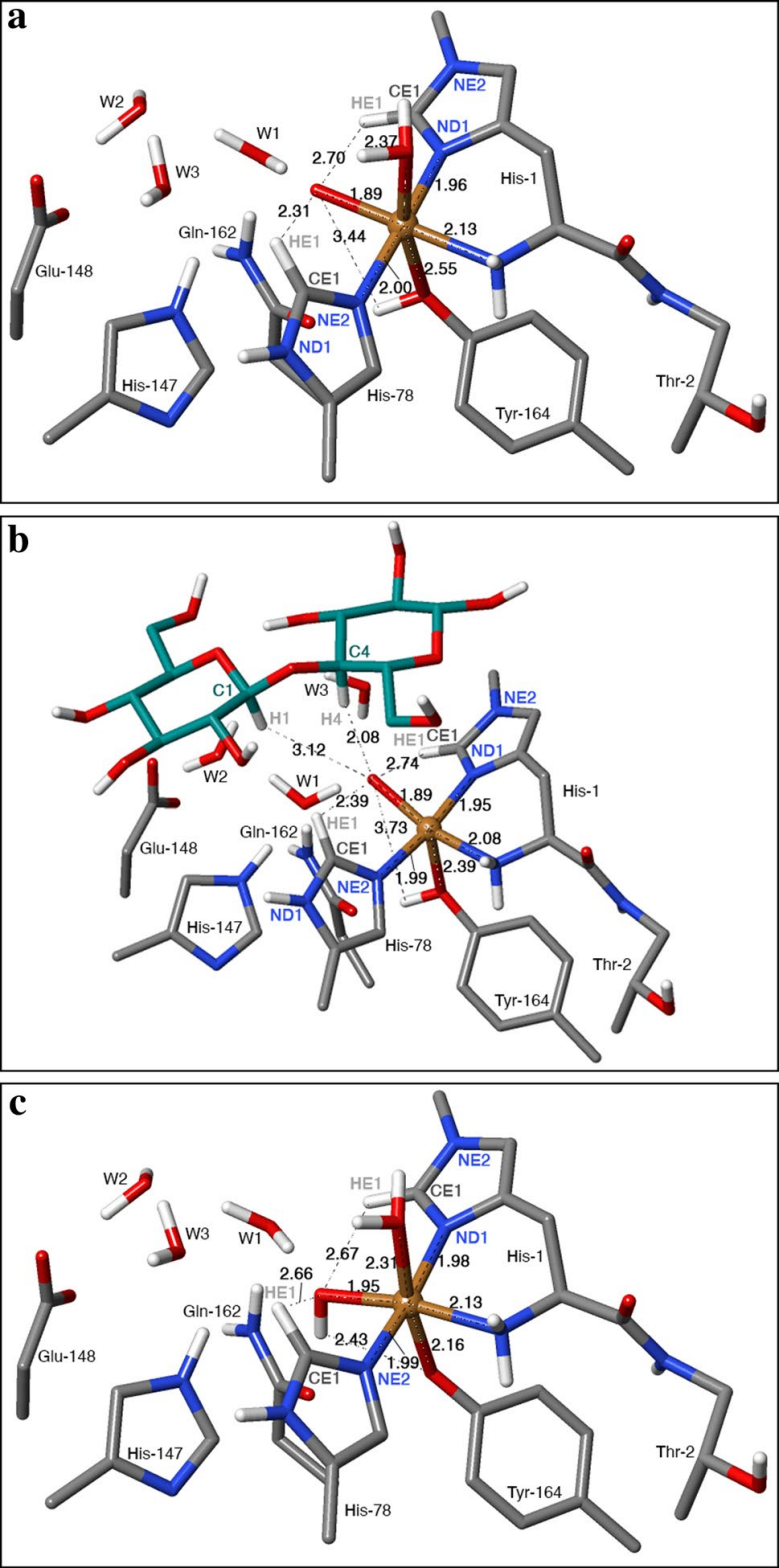
The active site of *Ls*AA9 LPMO **a** with [CuO]^+^ and without the substrate, **b** with [CuO]^+^ and the substrate, **c** with [CuOH]^2+^ and without the substrate. For clarity, non-polar hydrogen atoms are not shown

The QM system was slightly larger than in the previous study [35]. It consisted of the Cu ion, the oxy or OH ligand, the entire His-1 (coordinated to Cu by the amino-terminal group and the ND1 atom), all atoms in Thr-2, except the backbone O atom, the sidechain of His-78 (truncated at CA and coordinated to Cu by the NE2 atom), the sidechain of Tyr-164 (truncated at CA and coordinated to Cu by the OH atom), the sidechains of His-147, Glu-148 and Gln-162 (truncated at the CA atoms), as well as three water molecules. In the structure with the substrate, a disaccharide fragment was included in the QM system, whereas in the structure without the substrate, instead an additional water molecule coordinated to Cu. Structures with [CuOH]^+^ were generated by moving the proton on Tyr-164 to the oxy group. The QM system is illustrated in Fig. 2.

## Results

### Hydrogen-abstraction energies of an isolated histidine model

**Fig. 3:**
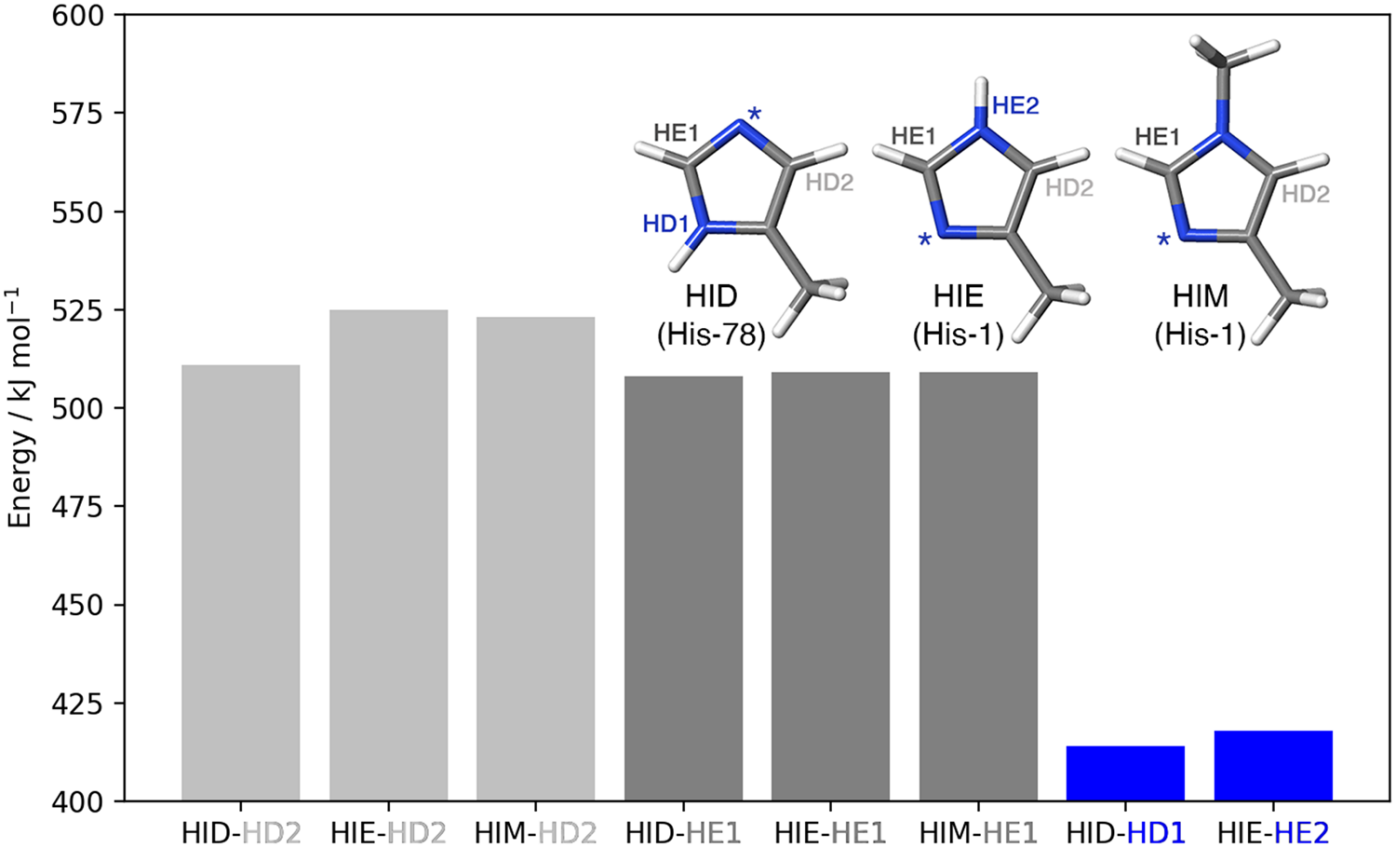
Hydrogen-abstraction energies (B3LYP/def2-TZVPP) for the various H atoms in the three models. The inset shows the three histidine models and the names of the abstracted H atoms. The star (*) marks which N atom coordinates to Cu in the active site

The two coordinating histidine residues in LPMOs display different bonding situations. To probe if this gives rise to different hydrogen-abstraction energies, we first calculated the QM energy for homolytic removal of each of the hydrogen atoms in the imidazole ring (HD2, HE1, HD1 and HE2) in three different models of the sidechain of an isolated histidine residue. The three models resemble different bonding situations in the LPMO active site (cf. Fig. 2 and the inset in Fig. 3): the HID model is protonated on the ND1 atom. This is a model of His-78 in LPMO, where NE2 binds to Cu. The HIE model is protonated on the NE2 atom, which would correspond to a non-methylated His-1 residue in LPMO (where ND1 binds to Cu). The third model (HIM) has a methyl group on NE2 (and ND1 is deprotonated). This corresponds to the methylated His-1 in *Ls*AA9, where ND1 coordinates to Cu. The calculated hydrogen-abstraction energies are presented in Fig. 3.

The effect of methylating the NE2 atom is rather small on the hydrogen-abstraction energy for the HD2 and HE1 atoms: for the HE1 atom, the hydrogen-abstraction energy increases by less than 1 kJ/mol; whereas for the HD2 atom, it increases by 12 kJ/mol for the HID model, while it decreases by 2 kJ/mol for the HIE model. The trends obtained with TPSS or a smaller basis sets are very similar (show in Table S1 in the Supporting Information). Notably, it is much easier to remove a hydrogen from the nitrogen atoms (414–418 kJ/mol) than from the carbon atoms (508–525 kJ/mol). This may indicate that NE2 atom of the amino-terminal residue is methylated to avoid that the HE2 atom is abstracted. Still, this does not explain why the other His ligand is not methylated. Further, the hydrogen atoms bound to N in the imidazole rings are far from the copper, meaning that abstraction of this atom is an unlikely first step in the reaction. Therefore, we study the abstraction of HE1 in His-1 and His-78 as a potential first step in the next subsection (these protons were selected because they are nearest to the [CuO]^+^ oxyl atom, cf. Fig. 2a, b).

### Hydrogen abstraction from histidine in the protein

The oxidation of the two histidine residues binding to Cu in the enzyme (His-1 and His-78 as is shown in Fig. 1) was investigated employing QM/MM, as described in the Methods section. We focus on [CuO]^+^ as the reactive species, since this moiety can be formed from reaction with H_2_O_2_, and H_2_O_2_ is known to be responsible for oxidative damage [30, 36]. We compare the results to the same reactions performed with a [CuOH]^2+^ intermediate. In this intermediate, the H atom in [CuOH]^2+^ is transferred from Tyr-164 to the oxyl group, i.e., [CuOH]^2+^ implies a deprotonated Tyr-164 ligand; cf. Fig. 2c. This species was previously proposed to have a protecting role if reduced LPMOs reacts with H_2_O_2_ in absence of substrate [35]. For both [CuO]^+^ and [CuOH]^2+^, we have studied the abstraction of the HE1 proton in His-1, as well as in His-78. We have also studied the abstraction of HE1 from His-1 without any methylation of NE2 of His-1. The calculations are performed for three spin states: the closed-shell singlet, the triplet and the open-shell singlet states. Since also the substrate is known to protect the enzyme against oxidative damage [15, 19, 55], we started from two different (previously optimised [35]) structures, one with and one without a bound trisaccharide substrate. Note that when the substrate is bound, the water ligand of the Cu ion trans to Tyr-164 is displaced (cf. Fig. 2a, b). Consequently, we have 3 × 2 × 2 × 3 = 36 separate reaction profiles that are described in the following. The results are collected in Table S2 and summarised in Table 1.

**Table 1:** Results for the hydrogen-abstraction reaction from His-1 or 78 in *Ls*AA9, showing only the best results obtained at the B3LYP/def2-TZVPD level (results for all spin states are shown in Table S2 in the Supporting Information)

O/OH	Substrate	His	**R**	**TS1**	**I**	∆*E*^#^	∆*E*
O	No	1_ M_	2.68	1.2	0.98	93	52
		78	2.33	1.2	**0.98**	100	**44**
		1_H_	2.71	1.1	**0.97**	91	**47**
	Yes	1_ M_	2.75	*1.2*	**0.98**	*142*	**63**
		78	2.39	*1.2*	0.98	*99*	104
		1_H_	2.77	*1.2*	**0.98**	*146*	**60**
OH	No	1_ M_	2.67				
		78	2.65	1.2	**1.02**	**112**	**105**
		1_H_	2.68				
	Yes	1_ M_	2.22				
		78	3.07	1.1	1.02	190	152
		1_H_	2.40				

The first column indicates if the reaction started from the [CuO]^+^ or [CuOH]^2+^ state. The second column shows whether the model involves the substrate or not. The third column shows whether the hydrogen atom was abstracted from His-1 with or without the methyl group (1_M_ or 1_H_) or from His-78. The following three columns show the O–H distance (in Å) for the reactant, transition state or intermediate. The last two columns show the activation and reaction energies in kJ/mol. For the latter five columns, results in regular style were obtained in the triplet state, those in italics were obtained for the closed-shell singlet and those in bold face were obtained for the open-shell singlet

We start by a discussion of the electronic structures of the [CuO]^+^ reactant state (**R**, shown in Fig. 2a, b). As discussed previously, based on calculations with smaller QM systems [26, 30, 31], the [CuO]^+^ reactant can best be described as Cu^2+^ (*d*^9^) coupled to a O^–^ radical. Thus, it has two unpaired electrons, which can either be ferromagnetically coupled to a triplet state or antiferromagnetically coupled to an open-shell singlet state. The larger QM system in our investigation does not change this interpretation: the spin population for the triplet reactant state is ~ 0.56 *e* on Cu and ~ 1.17 *e* on O (at the B3LYP/def2-TZVPD level), with the remaining spin on the direct ligands of Cu (not on the Tyr ligand). The substrate does not change this much (no spin is found on the substrate, nor on the water molecule that coordinates to Cu and is replaced by the substrate). In the open-shell singlet without the substrate, the spin on Cu is still ~ 0.6 *e*, but that on O has decreased to ~ 0.85 *e* (with the opposite sign). With the substrate, the two spin populations decrease to 0.31 *e* and ~ 0.42* e*, respectively.

At the B3LYP/def2-TZVPD level of theory, the [CuO]^+^ models are always most stable in the triplet state. Without the substrate, the closed-shell singlet state is 53–76 kJ/mol less stable and the open-shell singlet state is 13 kJ/mol less stable. With the substrate, the closed-shell singlet state is somewhat further destabilised (81–86 kJ/mol less stable than the triplet) and the open-shell singlet is 18–23 kJ/mol less stable than the triplet.

**Fig. 4:**
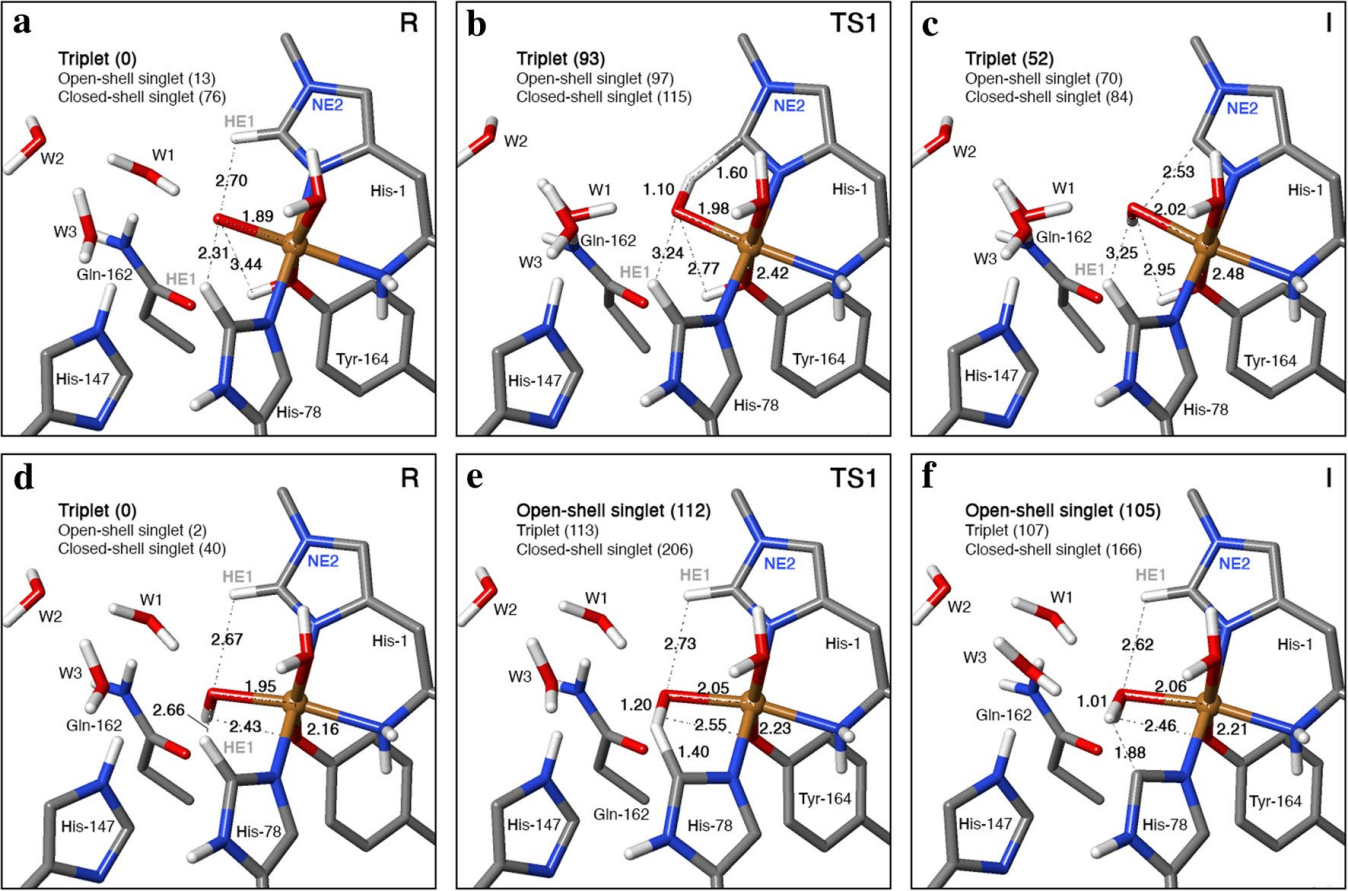
The hydrogen-abstraction reactions, illustrated for the systems without the substrate, in which [CuO]^+^ abstracts the HE1 proton from His-1 (**a**–**c**) or [CuOH]^2+^ abstracts the HE1 proton from His-78: **a** and **d** reactant states (**R**); **b** and **e** transition states (**TS1**) and **c** and **f** intermediates (**I**). The figures also show key distances in Å and energies in kJ/mol

The energies and structures for the H-abstraction reaction from His-1 by the [CuO]^+^ moiety is shown in Fig. 4a–c, using the structure without substrate and with methylated His-1 as example. A transition state (**TS1**) as well as a stable intermediate (**I**) with a Cu-bound OH group were located for the reactions with both His-1 and His-78. Without the substrate, the lowest transition state is found on the triplet surface, at a H–O distance of 1.1–1.2 Å (Table 1; results for all states are shown in Table S2), i.e. much closer to the intermediate (~ 1.00 Å) than the reactant (2.2–2.8 Å). The barrier is highest for His-78, 100 kJ/mol, slightly lower for His-1, 93 kJ/mol, and even lower for His-1 without the methyl group, 91 kJ/mol. With the substrate, the lowest barrier is found for the closed-shell singlet, but the triplet is only 2–10 kJ/mol higher. The transition state is still found for a H–O distance of 1.2 Å. The barrier is almost the same for His-78, 99 kJ/mol, but the barriers for His-1 are appreciably higher: 142–146 kJ/mol, and this time slightly higher for the His residue without the methyl group. The reaction energy is 44–52 kJ/mol without the substrate and 60–104 kJ/mol with the substrate. Thus, formation of intermediate **I** is always uphill.

In intermediate **I** for the reaction with [CuO]^+^ (Fig. 4c), the spin on Cu in the triplet state is still 0.54–0.67 *e*, whereas that on O in the formed OH group has decreased to 0.16–0.47 *e*, and the reacting His group has acquired almost one electron (0.99–1.14 *e* for His-1, but 0.78–0.86 *e* on His-78, mainly on the C atom that lost its proton, ~ 0.7 *e*). In the transition state (**TS1**), the situation is intermediate. The open-shell singlet **I** has ~ 0.57 *e* spin on Cu and 0.52–0.80 *e* on the reacting His residue with the opposite sign, but only 0.04–0.25 *e* on OH. The triplet and the open-shell singlet states are close in energy (within 2–18 kJ/mol), but the singlet is normally most stable. The closed-shell singlet is 24–34 kJ/mol less stable without the substrate and 57–114 kJ/mol with the substrate.

In the reactant state (**R**) without the substrate, the Cu ion is six-coordinate with short bonds to the two histidine sidechains (1.96–2.00 Å) and to the O ligand (1.89 Å), an intermediate Cu–N bond to the amino terminal (2.13 Å), and long Cu–O bonds to water (2.36–2.37 Å) and the (protonated) Tyr ligand (2.54–2.55 Å; cf. Fig. 2a). The substrate displaces the water ligand, leading to a five-coordinate Cu ion and slightly shorter bonds to all ligands (0.005–0.05 Å, but 0.15–0.16 Å for Tyr-164; Fig. 2b). In the intermediate, **I**, the Cu–O distance to the OH group has increased by 0.11–0.19 Å, reflecting its protonation (Fig. 4c; but only 0.03 Å for two complexes for which **I** is an open-shell singlet that gives shorter distances to Cu). The other ligands show rather small changes. The transition state is intermediate.

In the reactant state (**R**), the O atom of [CuO]^+^ forms a hydrogen bond to a water molecule (W1), which also receives a hydrogen bond from one of the HE2 atoms of Gln-162 (Fig. 2a). W1 also donates a hydrogen bond to another water molecule (W2), which is hydrogen bonded to one of the carboxylate atoms of Glu-148. For structures with the substrate, W1 also receives a hydrogen bond from HE2 of His-147 (Fig. 2b). The OE1 atom of Gln-162 receives a hydrogen bond from the OH group of Tyr-164. All these interactions are kept also in the reaction intermediate **I**.

We now turn to the reactant state with a [CuOH]^2+^ moiety (Figs. 2c, 4d–f). For this state, the triplet is still most stable, but only 1–2 kJ/mol more stable than the open-shell singlet without the substrate (7–13 kJ/mol with the substrate). The closed-shell singlet is 39–43 kJ/mol less stable. The [CuOH]^2+^ reactant state has a complicated electronic structure with ~ 0.62 *e* on Cu, only ~ 0.22 *e* on OH, but 0.78 (without) or 0.91 *e* (with substrate) on the deprotonated Tyr ligand in the triplet state. A similar spin distribution was observed also in a previous study (with a smaller QM region) and it fits experimental observations [35]. For the open-shell singlet, the spin populations are ~ 0.50 *e* on Cu, ~ 0.15 *e* on OH (aligned with Cu) and 0.81–0.88 *e* on Tyr (with the opposite sign). Thus, it is best described as Cu^2+^–OH^–^–TyrO^⋅^, but with some radical character on OH^–^.

Interestingly, hydrogen abstraction from His-1 (both with and without the methyl group) failed for all systems with [CuOH]^2+^—a potential-energy scan of the abstraction of the HE1 atom to the Cu-bound OH group was uphill by 108–159 kJ/mol and releasing the H–O bond distance restraint led to that the hydrogen atom returned to His-1. However, with His-78, a transition state was found (**TS1**), as well as a stable intermediate (**I**) with the HE1 hydrogen atom abstracted from histidine to a water bound to Cu^2+^ (illustrated in Fig. 4d–f without the substrate). The transition state is late with a H–O distance of 1.1–1.2 Å. The barrier for this reaction is 112 kJ/mol without the substrate and 190 kJ/mol with the substrate. The reaction energy is 105 kJ/mol without and 152 kJ/mol with the substrate. Thus, the [CuOH]^2+^ state does not seem to react with the active site histidine residues, supporting its role as a protective intermediate.

For the intermediate in the triplet state, the spin populations are 0.62–0.67 *e* on Cu, 0.10–0.11 *e* on O, 1.21–1.47 *e* on the Tyr ligand and 0.24–0.57 *e* on the deprotonated His ligand. The open-shell singlet has 0.63–0.87 *e* on Cu, 0.05–0.01 *e* on O, 0.63–0.87 *e* on the Tyr ligand (with the opposite sign) and 0.13–0.21 *e* on the deprotonated His ligand. Without the substrate, the triplet and the open-shell singlet states are degenerate within 2 kJ/mol. However, with the substrate, the triplet is 19 kJ/mol more stable. The closed-shell singlet is 61–67 kJ/mol less stable. The same applies for the transition state (**TS1**): the triplet and the open-shell singlet degenerate within 1–7 kJ/mol, whereas the closed-shell singlet is 69–93 kJ/mol less stable.

The deprotonated Tyr ligand gives a much shorter Cu–O bond in the reactant state (2.10–2.16 Å) than when it is protonated (2.39–2.55 Å). On the other hand, the OH group gives a 0.04–0.06 Å longer Cu–O bond (1.92–1.95 Å) than the O group. This gives rather small changes in the Cu–N distances (Fig. 2). In the [CuOH]^2+^ state, the proton on Tyr-164 has moved to the OH ligand, but it keeps the hydrogen bond to Gln-162 (Fig. 2c). All these interactions are kept in the reaction intermediate, **I**.

Since the [CuOH]^2+^ reactant state (**R**) is formed from the [CuO]^+^ state by a proton transfer from the Tyr hydroxide group, energies of these two states are comparable for all models. For the reactant state, the [CuOH]^2+^ state with a deprotonated Tyr is actually always most stable, by 45–79 kJ/mol. The difference decreases to 15 kJ/mol for the intermediate (**I**) with the substrate, and the state with a protonated Tyr is actually most stable by 16 kJ/mol for the intermediate (**I**) without the substrate.

### Rebound reaction

In the previous section, we showed that [CuO]^+^ can abstract the HE1 from either His-1 or His-78 with a barrier of 91–146 kJ/mol and a reaction energy of 44–104 kJ/mol. In this section, we consider the rebound of the formed OH group in the intermediate **I** to the histidine radical. Again, we studied both [CuO]^+^ or [CuOH]^2+^ (which have become [CuOH]^+^ and [CuOH_2_]^2+^ for the intermediate), active sites with or without substrate, reactions with either His-78 or His-1 (the latter either with or without the methyl group), as well as either triplet, open-shell singlet and closed-shell singlet states. This gives in total 24 reactions, the results of which are listed in Table S2 and summarised in Table 2.

**Table 2:** Results for the rebound reaction of the Cu-bound hydroxyl or water group to His in LPMO, showing only the best results obtained at the B3LYP/def2-TZVPD level (results for all spin states are shown in Table S3 in the Supporting Information)

O/OH	Substrate	His	**I**	**TS2**	**P**	∆*E*^#^	∆*E*
O	No	1_ M_	2.34	1.6	*1.33*	49	− *287*
		78	**2.27**	1.7	*1.31*	33	− *245*
		1_H_	**2.33**	1.6	*1.33*	36	− *291*
	Yes	1_M_	**2.96**	1.9	*1.33*	99	− *271*
		78	2.53	1.8	*1.33*	34	− *363*
		1_H_	**3.03**	1.9	*1.35*	93	− *267*
OH	No	78	**2.76**	1.8	*1.33*	164	− *222*
	Yes	78	3.35	1.8	*1.33*	138	− *347*

The first column indicates if the reaction was started from the [CuO]^+^ or [CuOH]^2+^ state (i.e. if the intermediate contains [CuOH]^+^ or [CuOH_2_]^2+^). The second column shows whether the model involves the substrate or not. The third column shows whether the hydrogen atom was abstracted from His-1 with or without the methyl group (1_M_ or 1_H_) or from His-78. The following three columns show the O–C distance (in Å) for the intermediate, transition state or product. The last two columns show the activation and reaction energies in kJ/mol (relative to the intermediate **I**). For these five columns, results in regular style were obtained in the triplet state, those in italics were obtained for the closed-shell singlet and those in bold face were obtained for the open-shell singlet

**Fig. 5:**
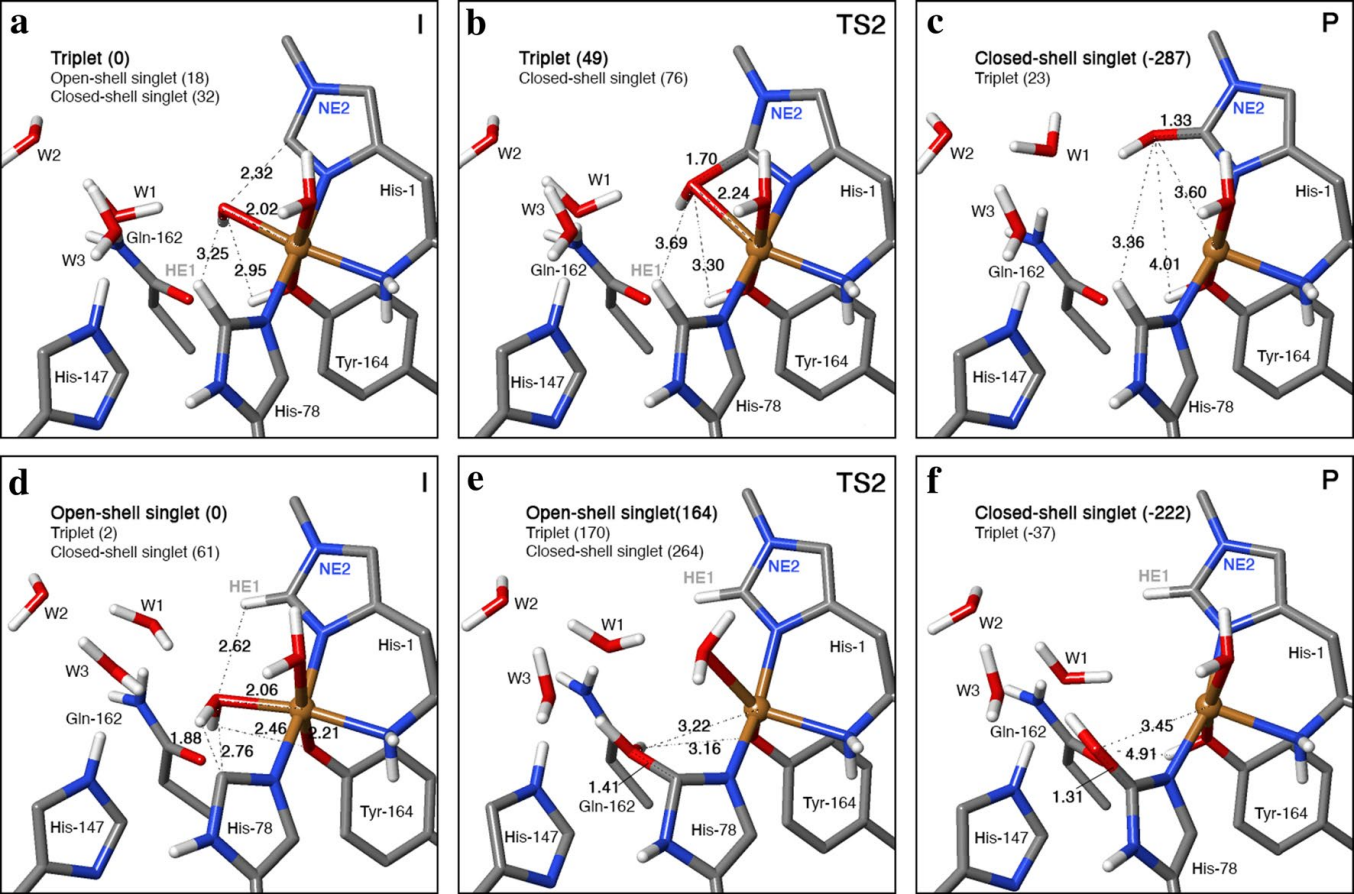
The rebound reaction, illustrated for the systems without the substrate, in which OH from [CuOH]^+^ rebounds to CE1 of His-1 (**a**–**c**) or OH_2_ from [CuOH_2_]^2+^ rebounds to CE1 of His-78 (and one proton goes to Tyr-164): **a** and **d** intermediates (**I**); **b** and **e** transition states (**TS2**) and **c** and **f** products (**P**). The figures also show selected distances in Å. All energies are in kJ/mol

The reaction starts from the intermediate (**I**) and goes via a transition state (**TS2**) to a product (**P**), which contains a hydroxylated histidine ring with OH on either His-78 or His-1: the C–O bond length is 1.31–1.35 Å in the product (**P**) compared to 2.27–3.35 Å in the starting intermediate (**I)**—structures of the intermediate **(I**), transition state (**TS2**) and product (**P**) are displayed in Fig. 5. The product is always most stable in the closed-shell singlet state, i.e. with the Cu ion in the + I oxidation state. When the reaction is started from [CuO]^+^, the triplet state for the product is 245–362 kJ/mol less stable than the closed-shell singlet and the open-shell singlet state is not found.

When the reaction is started from [CuOH]^2+^, the intermediate has a copper-bound water molecule, which cannot directly rebind to the histidine radical. Instead, one of the protons needs to move to another group, which complicates the reaction. The most stable product is the closed-shell singlet with a hydroxylated histidine and the proton moved back to Tyr-164 (i.e. the same state as for the reactions started from [CuO]^+^; cf. Fig. 5f). However, other products can also be found, especially on the triplet surface, e.g. with C=O or CH(OH) groups on the histidine or with the proton on Gln-162, which is the closest proton acceptor. Yet, these products are always appreciably less stable, by 158–221 kJ/mol for the triplet structures and by 24–43 kJ/mol for the alternative protonation states.

In the product state (**P**) without the substrate, the hydroxyl group on His-1 forms a hydrogen bond to W1 (Fig. 5c). With the substrate, it instead receives a hydrogen bond from W1 and donates a hydrogen bond to W3. The hydroxyl group on His-78 donates a hydrogen bond to W1 (without the substrate; Fig. 5f) or to the substrate.

In all cases without the substrate, the Cu ion remains five-coordinated in the product state, but the bonds to the water molecule and to Tyr-164 are long, 2.32–2.36 and 2.50–2.57 Å, respectively. The bond to the amino terminal is intermediate, 2.15–2.20 Å, whereas the two bonds to the histidine imidazole rings are short, 1.95–1.98 Å. With the substrate, the Cu ion is four-coordinated and the bonds are slightly shorter: 1.90–1.94 Å to histidine, 2.10–2.12 Å to the amino terminal and 2.31–2.45 Å to Tyr-164.

The transition state for the rebound reaction is always found on the triplet surface at a C–O distance of 1.6–1.9 Å (but 1.4 Å when Tyr is deprotonated; cf. Table 2). The barriers are varying, 33–99 kJ/mol for the reactions started from [CuO]^+^ and 138–164 kJ/mol for the reactions started from [CuOH]^2+^ (relative to the intermediate). Removing the methyl group on His-1 reduces the activation energy by 6–13 kJ/mol, showing some protection by the methyl group.

## Discussion

**Fig. 6:**
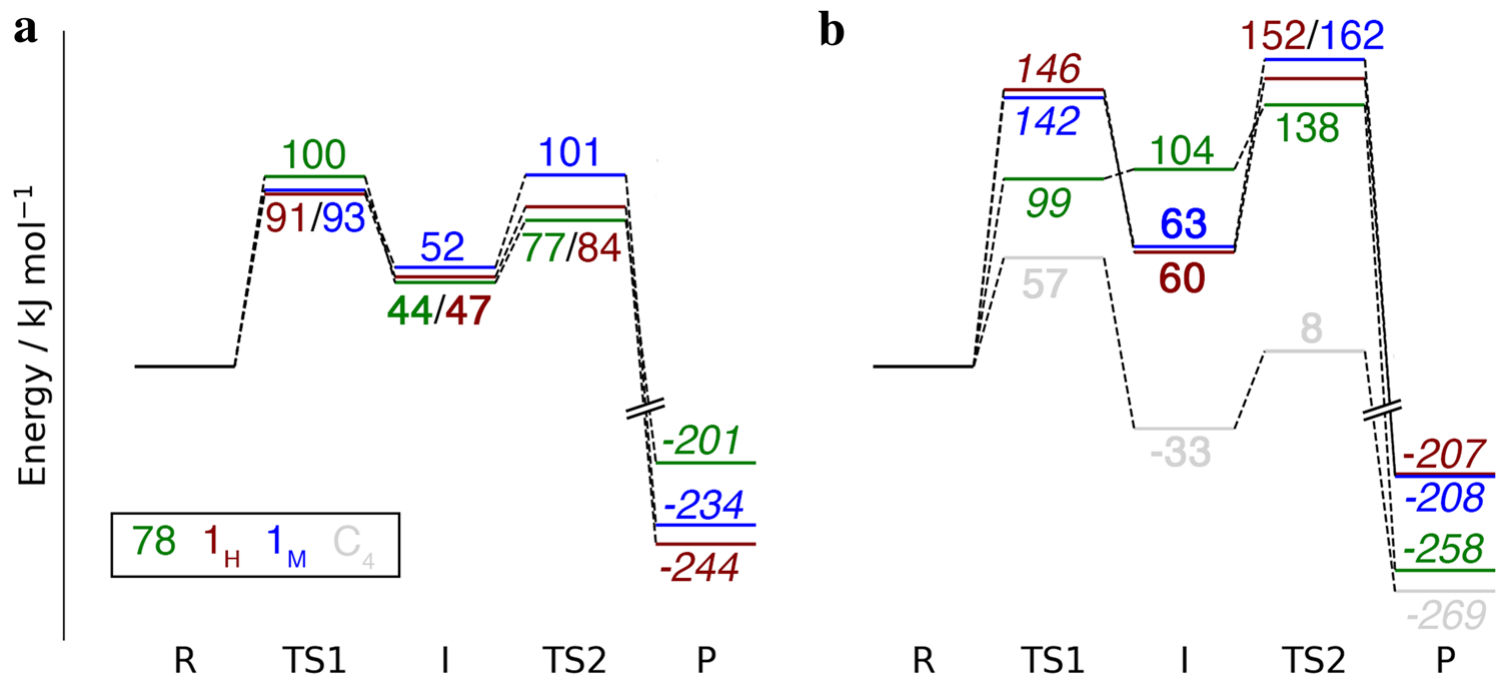
Energy diagrams for the hydrogen-abstraction and rebound reactions in *Ls*AA9 started from [CuO]^+^, without (**a**) and with the substrate (**b**), shown for the best results obtained at the extrapolated B3LYP/def2-TZVPD level. The reference is the reactant state (**R**) . The colours indicate whether the hydrogen atom was abstracted from His-1 with or without the methyl group (blue and red, respectively) or from His-78 (green). The energies for the hydrogen-abstraction from the C4 atom of the substrate are shown in grey. All energies are given in kJ/mol. Results in regular style were obtained in the triplet state, those in italics were obtained for the closed-shell singlet and those in bold face were obtained for the open-shell singlet

In Fig. 6, we summarise the results for both reactions (hydrogen abstraction and rebound) for the lowest spin state for the six reactions started from [CuO]^+^ and putting all reactants at the same energy level (with the reactant state, **R**, as the reference). Both with and without the substrate, the methyl group on His-1 increases the highest of the two reaction barriers by 10 kJ/mol (from 91 to 101 kJ/mol without and from 152 to 162 kJ/mol with the substrate; the rate-limiting step also changes from hydrogen abstraction to rebound. This shows that the methyl group in fact protects His-1 from self-oxidation. The difference may seem small, but it corresponds to an increase in the estimated turnover time (*k*_cat_^–1^) from 0.3 to 18 h (estimated with classical transition-state theory [56] and a pre-exponential constant of *k*_B_*T*/*h* = 6.2 × 10^12^/s). Moreover, the oxidation of unmethylated His-1 has a lower maximum barrier (91 kJ/mol) than that of His-78 (100 kJ/mol), but the oxidation of methylated His-1 has essentially the same barrier (101 kJ/mol), explaining why there is no need to methylate also His-78. With the substrate, the maximum barrier for hydroxylation of His-78 is smaller than that for His-1 both with and without methylation, but all barriers are so large that no reaction is expected (*k*_cat_ < 5 × 10^–12^/s). Likewise, the [CuOH]^2+^ state leads to barriers that are too high for any reaction to occur, both with and without substrate (269–290 kJ/mol).

This fits well with the recent suggestion that the [CuOH]^2+^ state may protect the active site from histidine oxidation, based on experimental and theoretical characterisation of a tyrosine radical [32–35]. Further, an intermediate with a characteristic signature of a histidine radical was very recently observed and characterised by HERFD-XAS and UV–Vis spectroscopy [57]. It was suggested that this intermediate decays to an intermediate with a tyrosine radical, which was found to be inactive with respect to substrate oxidation. These result fit well with the mechanism proposed here, in which the histidine radical intermediate **I** is formed from the reactive [CuO]^+^ species. The formation of **I** is uphill, and **I** was indeed found to be short lived [57].

Finally, we also note that a lower barrier (57 kJ/mol) is calculated for the abstraction of the hydrogen at C4 from the substrate by [CuO]^+^ (grey path in Fig. 6b; further results are given in the Supporting Information). This shows that the substrate is much more prone to be oxidised than the His ligands. We have not investigated whether a methylation of His-1 affects the reaction energy of the substrate C–H abstraction, but previous QM-cluster results and our calculations on the isolated histidine residues suggest that this is not the case [26].

We can, thus, conclude that a self-oxidation mechanism involving [CuO]^+^ abstracting a hydrogen from His-1 is consistent with the observation that methylation of His-1 makes the deactivation less likely. It is known that histidine residues susceptible to oxidative damage in some other metalloenzymes are typically converted to 2-oxo histidine [58, 59]. The product** P** contains 2-hydroxy-histidine and it is possible that it may further react to 2-oxo histidine. Our results for isolated histidine highlights that the most susceptible bonds in the imidazole ring are the N–H bonds. Thus, any further reaction to 2-oxo histidine may involve breaking of the NE2–H bond in His-1, and we are currently investigating this possibility.

## Conclusions

We have studied oxidation of the histidine ligands in LPMO, investigating the hypothesis that methylation of the His-1 residue, observed in many families of LPMO, may protect against such oxidation. We have considered oxidation of both His-1 and His-78, with both [CuO]^+^ and [CuOH]^2+^ as the reactive state, as well as reactions for the enzyme both with and without a bound substrate. We make several interesting observations.

- The reaction consists of an initial hydrogen abstraction of the HE1 proton, followed by rebound of the formed OH or OH_2_ group.
- The intermediate is appreciably less stable than the reactant state (by 44–104 kJ/mol for [CuO]^+^ and 105–152 kJ/mol for [CuOH]^2+^).
- The product is 117–258 kJ/mol more stable than the reactant state.
- The activation energy for the hydrogen abstraction and the rebound step are similar for [CuO]^+^, but the latter is much higher for [CuOH]^2+^ (with the reactant as the reference state).
- The maximum activation energy is 91–101 kJ/mol for [CuO]^+^ without the substrate, corresponding to rates of 0.06–4 h^–1^. For the other cases, the barriers are higher, making the reactions very slow.
- Methylation of His-1 reduces the maximum activation barrier by 10 kJ/mol both with and without the substrate. Without the substrate, methylated His-1 has a similar maximum barrier as His-78, increasing the estimated lifetime of the enzyme from 0.3 to 18 h.
- The reactions have complicated spin properties with sometimes close-lying triplet, open-shell singlet and closed-shell singlet states. They start from a triplet state and the product is a closed-shell singlet. The intermediate is an open-shell singlet or a triplet.

Our results point out two tentative reasons why His-1 is methylated. First, the simple model calculations indicate that the methylation avoids hydrogen abstraction of the HE2 atom (which is replaced by the methyl group); the bond-dissociation energy of N-bound hydrogen atoms is ~ 100 kJ/mol lower than that of C-bound hydrogen atoms. However, this atom is too far from the Cu-bound O group (4.7–5.0 Å) to allow a direct reaction, studied by the current methods. Second, methylation increases the maximum barrier for oxidation of His-1 by 10 kJ/mol, viz. to the same level as for His-78 and increasing the lifetime of the enzyme 60-fold. We believe that the second reason is more important and also explains why only His-1 and not His-78 is methylated. This nicely illustrates how QM calculations may explain the design of enzymes.

## Data Availability

Raw data from the calculations are available from the corresponding author upon request.
